# Identifying patients at low risk for atrial fibrillation after cryptogenic stroke: the PAVA score

**DOI:** 10.1093/europace/euag183

**Published:** 2026-09-03

**Authors:** Baptiste Maille, Noémie Resseguier, Eloi Marijon, Nathalie Behar, Marc Badoz, Fabrice Vuillier, Cecile Malrain, Claire Grygorowicz, Victor Klein, Aymeric Menet, Maryam Dridi, Christelle Haddad, David Calvet, Laurent Suissa, Luciano A Sposato, Jean-Claude Deharo, Yannick Bejot, Charles Guenancia

**Affiliations:** Rhythmology Department, CHU La Timone, 264 Rue Saint-Pierre, 13005 Marseille, France; CEReSS-Health Service Research and Quality of Life Center, School of Medicine, Aix-Marseille University, Marseille, France; Division of Cardiology, European Georges Pompidou Hospital, Paris, France; Rennes University Hospital, Department of Cardiology, Rennes, France; Cardiology Department, CHU Besançon, Besançon, France; Neurology Department, CHU Besançon, Besançon, France; Rennes University Hospital, Department of Neurology, Rennes, France; Université Bourgogne Europe, CHU Dijon Bourgogne, Service de Cardiologie, EA7460 PEC2, 21000 Dijon, France; Rhythmology Department, CHU La Timone, 264 Rue Saint-Pierre, 13005 Marseille, France; Cardiology Department, Groupe Hospitalier Institut Catholique de Lille, Lille, France; Cardiology Department, Groupe Hospitalier Institut Catholique de Lille, Lille, France; Department of Electrophysiology, Hôpital Cardiologique Louis Pradel, Hospices Civils de Lyon, 28 avenue du Doyen Lepine, Bron 69500, France; Department of Neurology and Stroke Unit, GHU-Paris Psychiatrie et Neurosciences, Hôpital Sainte-Anne, Université de Paris Cité, INSERM U1266, Institute of Psychiatry and Neuroscience, Paris, France; Department of Neurology and Stroke Unit, CHU La Timone, Marseille, France; Department of Clinical Neurological Sciences, Schulich School of Medicine and Dentistry, Western University, London, Ontario, Canada; Rhythmology Department, CHU La Timone, 264 Rue Saint-Pierre, 13005 Marseille, France; Université Bourgogne Europe, CHU Dijon Bourgogne, Service de Neurologie , EA7460 PEC2, 21000 Dijon, France; Université Bourgogne Europe, CHU Dijon Bourgogne, Service de Cardiologie, EA7460 PEC2, 21000 Dijon, France

**Keywords:** Stroke, ESUS, Prediction, Cryptogenic stroke, Atrial fibrillation

## Abstract

**Aims:**

Continuous cardiac monitoring with implantable loop recorders (ILRs) is recommended after cryptogenic stroke to detect occult atrial fibrillation (AF). We aimed to develop and validate a dedicated risk score for AF detection in patients undergoing ILR monitoring after cryptogenic stroke.

**Methods and results:**

This multicentre study enrolled 1002 consecutive patients (mean age 66.0 ± 12.3 years, 57.9% male) who received an ILR after cryptogenic stroke. The primary outcome was a first episode of AF or atrial tachycardia (AT). A score was developed in a randomly split development cohort and validated internally, accounting for competing risks, missing data imputation, and hazard ratio-based weighting. Among the 668 patients in the development cohort, cumulative incidences of AF/AT at 1, 2, and 3 years were 20.3%, 28.9%, and 33.0%, respectively. Four independent predictors were identified: premature atrial contractions > 200/24 h on Holter-ECG (2 points), age >58–78 years (3 points) or > 78 years (4 points), significant valvular heart disease (1 point), and left atrial dilatation (2 points), forming the PAVA (premature atrial contractions, age, significant valvular heart disease, and left atrial dilatation) score. Discrimination was good in both the development and validation cohorts (c-index 0.73 and 0.79, respectively). The 3-year areas under the curve were 0.800 and 0.905, respectively. A PAVA score ≤ 2, observed in 23.6% of patients, yielded negative predictive values of 89.2% and 100% in the development and validation cohorts. ROC-curve analysis confirmed superior clinical utility over existing scores (Brown ESUS-AF [Brown embolic stroke of undetermined source-atrial fibrillation], HAVOC).

**Conclusion:**

A PAVA score ≤ 2 demonstrates a high negative predictive value for AF/AT on ILR after cryptogenic stroke, offering a practical tool to optimize patient prioritization for ILR implantation.

What’s new?

- The PAVA score is the first risk score developed and internally validated specifically for the prediction of atrial fibrillation or atrial tachycardia (AF/AT) detected by implantable loop recorder (ILR) after cryptogenic stroke, in a multicentre cohort of 1002 consecutive patients, with a competing-risk framework accounting for death. The score combines four readily available variables from the routine post-stroke workup: premature atrial contractions > 200/24 h on Holter ECG (2 points), age 58–78 years (3 points) or > 78 years (4 points), significant valvular heart disease (1 point), and left atrial dilatation (2 points). Discrimination was good in both the development and validation cohorts (c-index 0.74 and 0.79; 2-year area under the curve 0.819 and 0.863, respectively), and was confirmed after multiple imputation of missing data and in an external cohort from two additional centres. A PAVA score ≤ 2 identified 23.6% of patients as being at low risk of AF/AT, with negative predictive values of 89.2% in the development cohort and 100% in the validation cohort. Decision-curve analysis showed greater clinical utility of the PAVA score than the Brown ESUS-AF and HAVOC scores, which misclassified as low risk a substantial proportion of patients who subsequently developed AF/AT. The PAVA score may therefore help prioritize ILR implantation in a context of constrained monitoring resources.

## Introduction

Cryptogenic stroke accounts for ∼ 20% of all ischaemic strokes. Depending on the screening method employed, up to 30% of atrial fibrillation cases are identified in this population.^1–3^ Based on these results, implantable loop recorders (ILRs) are now widely implanted after cryptogenic stroke, and recent European guidelines recommend their use as a first intention vs. prolonged external monitoring^4–6^ although significant variability in ILR implantation practices for atrial fibrillation (AF) detection after stroke persists across European centres.^7^

Although ILR implantation is generally considered safe,^8^ implantation is costly and time-consuming. In addition current guidelines and future projections of the number of patients who would be eligible for such diagnosis procedures at a national level pointed out a threat regarding the sustainability of health care system to meet the demand.^9^ Therefore, selection of patients with the highest likelihood of AF remains critical. A recent European Society of Cardiology (ESC) Working Group position paper^10^ and the latest European guidelines for the management of AF^4^ emphasized the need for patient selection criteria for ILR implantation after cryptogenic stroke by determining the preimplantation probability of AF. Previous scores for predicting AF after cryptogenic stroke have been published. Most are based on external ECG monitoring (repeated 12-lead ECG, 24-h Holter, and, rarely, prolonged external loop recorder), and thus did not include ILR-detected AF,^11–13^ although the Brown embolic stroke of undetermined source-atrial fibrillation (Brown ESUS-AF) score was partially developed based on AF detected by ILR monitoring.^14^ PROACTIA was the first score developed specifically for ILR-detected AF, in 236 patients with cryptogenic stroke, but it has not yet been validated.^15^ Despite the limited diagnostic accuracy of these scores,^16^ the ESC Working Group proposed a HAVOC score ≥ 4 or Brown ESUS-AF score ≥ 2 as the criterion for ILR implantation after cryptogenic stroke.^10^ However, two subsequent studies demonstrated limited performance of these scores in selecting cryptogenic stroke patients at high risk for AF for ILR implantation due to their lack of sensitivity.^17,18^

The aim of the present study was to develop and validate a dedicated clinical score to estimate the risk of AF after cryptogenic stroke in patients undergoing ILR monitoring.

## Methods

### Study design and population

This French multicentre cohort study was conducted at five university hospitals (Marseille, Dijon, Besançon, Paris, Rennes). In a second step of the study, patient data were collected from two other university hospitals (Lille and Lyon), forming an external validation dataset. It included all consecutive patients without previous AF who received an ILR after cryptogenic stroke between April 2015 and April 2022. All patients had a detailed initial workflow and a minimum follow-up of 6 months after ILR implantation. Patients were identified from prospectively built databases from implantations or retrospectively using the International Classification of Diseases (ICD-10) diagnostic codes for stroke diagnosis and Common Classification of Medical Procedures codes for ILR implantation. The diagnostic workflow included, at a minimum, cerebral and angio (cervical and intracranial arteries) computed tomography, brain magnetic resonance imaging, carotid ultrasound, transthoracic and/or transoesophageal echocardiography, and at least 24 h of ECG monitoring. After the complete diagnostic aetiological workup, cryptogenic strokes were considered, according to the TOAST classification.^5^ Patients with transient ischaemic attacks were not included. The study was approved and registered by an institutional ethic and scientific committee (CSE24-4) and is registered in the French National Health Data Hub (19352074).

### Baseline data

Baseline clinical data, stroke characteristics (date of stroke and related TOAST [Trial of ORG 10172 in Acute Stroke Treatment] classification; infarct location) and cardiovascular work-up results were collected from medical records. Obesity was defined as a body mass index (BMI) > 30 kg/m^2^.^19^ Data from transthoracic or transoesophageal echocardiography performed during the stroke diagnosis workup were included: left ventricular ejection fraction; presence of significant valvular heart disease (VHD) (defined as any moderate, or severe aortic and/or mitral stenosis or regurgitation)^20^; left ventricular hypertrophy; and presence of patent foramen ovale with or without septal aneurysm and left atrial (LA) dilatation. Left atrial dilatation was defined according to current guidelines, as an LA indexed volume ≥ 35 mL/m^2^ or when indexed volume was unavailable by an LA surface area > 20 cm^2^ or an increased LA diameter (>41 mm for men and > 39 mm for women).^21^

B-type natriuretic peptide (BNP) or *N*-terminal pro-B-type natriuretic peptide (NT-proBNP) value at the time of the stroke was collected. The number of premature atrial complexes (PAC) and presence of repetitive PAC (from three repetitive atrial beats) were retrieved from the 24-h Holter ECG. As previously published, we defined excessive supraventricular ectopic activity as PAC > 200/24 h^22^ (see Supplementary material online, *Figure S1A*). HAVOC^13^ and Brown ESUS-AF^14^ scores were calculated when possible.

### Outcome

Patients were followed for at least for 6 months after ILR implantation. End of follow-up corresponded to the last remote monitoring report or the last regular device physical interrogation according to practices at each centre. Atrial fibrillation was defined as an episode of irregular heart rhythm, without detectable *P*-waves, automatically detected by ILR, according to embedded algorithms. Occurrence of sustained (>30 s) atrial tachycardia (AT) or atrial flutter was considered and treated as AF episodes. Episodes of AF/AT were systematically reviewed and validated by senior electrophysiologists. The primary outcome was a first episode of AFAT exceeding 30 s. Death was considered a competing risk for AF during follow-up.

### Statistical analysis

A descriptive analysis of the data was performed based on the overall cohort. Quantitative data are expressed as mean (standard deviation [SD]) or median (quartile [Q]1, Q3). Qualitative data are expressed as count and percentage.

The prognostic model for AF was built as follows: first, the overall cohort was randomly split into two sub-cohorts, the development cohort (two-thirds of the overall cohort) and the validation cohort (one-third of the overall cohort); second, a prognostic model was developed and validated based on the development and validation cohorts, respectively.

The occurrence of AF may be precluded by death, creating a context of competing risk.^23^ The analysis therefore accounted for this potential bias.^24^ The analysis was limited to the first occurring event in the competing risks framework using the following quantities commonly used to summarize outcomes by event type: (1) the cause-specific hazard function, which for AF can heuristically be thought of as the probability of AF during a small interval of time, given that no death occurred before; and (2) the cumulative incidence function, which for AF corresponds to the probability of AF in the presence of competing death events. The delay from implantation of the ILR to the first observed event was calculated for each patient. In the case of no events during follow-up, observations were censored at the date of last follow-up.

To build the prognostic model for AF, univariate analyses were performed on the development cohort to identify variables associated with AF detection. Subdistribution hazard ratios (HRs) and their 95% confidence intervals (CIs) were estimated using the Fine and Gray model.^25^

For development of the score, numeric age values were converted to binary values using calculated best age cutoffs, because the lowest was related to a < 10% incidence rate of AF/AT and the highest to a specificity exceeding 90% (see Supplementary material online, *Figure S1B*). Variables with *P* values < 0.1 were included in the multivariable model. Their effect was studied in the multivariable model, to which data described in the literature were added. Weights were then attributed to each prognostic factor according to the coefficient value in the multivariable model to calculate the score using a logarithmic transformation of the HR.

The predictive accuracy of the score was assessed by studying the adapted c-index^24,26^and area under the receiver-operating characteristic (ROC) curve with their 95% CI. Moreover, patients were classified according to three groups (low, medium, and high score values). Then, cumulative incidences for AF were estimated in each of these subgroups and compared using the Gray test.^27^ Sensitivity, specificity, positive, and negative predictive values associated with low and high score-value groups were estimated with their 95% CI. Area under the ROC curve of the developed score was compared with those of the Brown ESUS-AF and HAVOC scores. The predictive accuracy of the score was analysed in the validation cohort using the same strategy.

As a first sensitivity analysis, the score was also calculated using the coefficients obtained in the multivariable model, leading to an unweighted score. Missing data points are inherent to large population studies. Complete case analysis was adopted for the model development. As a second sensitivity analysis, missing data on prognostic factors were imputed using the algorithm for multiple chained equations.^28–30^ Finally, the score was secondarily evaluated in a new cohort of patients enrolled in two other centres (Lille and Lyon). This second dataset was dedicated to an external validation of the score. The predictive accuracy of the score derived from the three sensitivity analyses was estimated.

All tests were two-sided. A *P* value < 0.05 was considered significant. Statistical analysis was performed using R version 4.2.2 (R Foundation for Statistical Computing, Vienna, Austria). The R package cmprsk was used for competing risk analysis and the R mice package was used for multiple imputation.

## Results

### Baseline characteristics

During the study, 1002 patients (mean age 66.0 [12.3] years) underwent ILR implantation for cryptogenic stroke at the five participating centres. Median (Q1, Q3) CHA_2_DS_2_-VASc score was 4 (3–5), Brown ESUS-AF score was 1 (0–2), and HAVOC score was 2 (0–4). The development and validation samples were similar regarding their baseline characteristics (see Supplementary material online, *Table S1*). Median (Q1, Q3) time from stroke to ILR implantation was 80 (36–194) days. Fourteen (1.4%) patients died during follow-up. After a mean (SD) and median (Q1, Q3) follow-up of 610 (442) and 518 (287–872) days, 276/1002 (27.6%) patients experienced at least one episode of AF, with a mean and median of 265 (292) and 149 (51–400) days after ILR implantation. At 1, 2, and 3 years, cumulative incidences of AF/AT were 20.3%, 28.9%, and 33.0%, respectively.

### Construction of the PAVA score

#### Univariable analysis

Atrial fibrillation/AT was found in 174 (26.0%) patients in the development cohort, occurring a mean of 272 (308) days after ILR implantation. The results of the univariate analysis, according to AF/AT incidence rate, are shown in *Table 1*. Age and 24-h PAC burden were continuously associated with AF/AT incidence rate, as well as the selected cutoffs. Previous hypertension, hypercholesterolaemia, LA dilatation, and significant VHD were also associated with AF/AT incidence rate.

**Table 1:** Univariable analysis according to onset of AF/AT at the end of follow-up (development cohort)

Variable	Development cohort (*n* = 668)	Hazard ratio (95% confidence interval)	*P* value
Sex (male)	387 (57.9%)	1.23 (0.91–1.66)	0.16
Age (years)			<0.001
Mean (SD)	65.8 (12.2)	1.06 (1.04–1.07)	<0.001
≤58 years	169 (25.2%)		
>58 and ≤78 years	414 (62.0%)	3.83 (2.25–6.51)	<0.001
>78 years	85 (12.7%)	7.40 (4.10–13.37)	<0.001
Infarct location			0.26
Supra-tentorial	462 (71.5%)		
Infra-tentorial	151 (23.4%)	0.76 (0.52–1.12)	0.17
Both	33 (5.1%)	1.24 (0.68–2.23)	0.48
BMI (kg/m^2^)			0.77
Mean (SD)	26.2 (4.8)	1.00 (0.97–1.04)	0.83
Obese (BMI > 30 kg/m^2^)	109 (17.8%)	0.95 (0.61–1.49)	0.83
Hypertension	392 (58.7%)	1.42 (1.04–1.94)	0.03
Hypercholesterolaemia	263 (39.4%)	1.63 (1.21–2.20)	0.001
Diabetes mellitus	100 (15.0%)	0.89 (0.57–1.37)	0.59
Active smoker	216 (32.3%)	0.85 (0.62–1.18)	0.34
Alcohol intake	44 (9.9%)	0.77 (0.40–1.48)	0.43
Obstructive sleep apnoea	45 (6.8%)	0.92 (0.53–1.60)	0.77
Peripheral arteriopathy or coronary artery disease	119 (17.8%)	1.27 (0.88–1.83)	0.21
Active neoplasia	12 (2.0%)	1.20 (0.40–3.68)	0.74
Heart failure	12 (1.8%)	1.14 (0.38–3.47)	0.82
Left atrial dimensions			
Left atrial indexed volume (mL/m^2^)	31.1 (12.3)	1.04 (1.03–1.05)	<0.001
Left atrial dilatation	189 (35.4%)	2.38 (1.72–3.30)	<0.001
Significant valvular heart disease	49 (7.9%)	2.73 (1.80–4.15)	<0.001
Left ventricular hypertrophy	100 (18.8%)	1.47 (0.99–2.18)	0.06
Patent foramen ovale	115 (19.7%)	0.64 (0.41–1.02)	0.06
NT-proBNP (pg/mL)	587 (2074)	1.00 (1.00–1.00)	0.91
PAC > 200/24 h	100 (26.6%)	2.83 (1.94–4.12)	<0.001
Burden of PAC/24 h	468 (1338)	1.00 (1.00–1.00)	<0.001

AF, atrial fibrillation; AT, atrial tachycardia; BMI, body mass index; NT-proBNP, *N*-terminal pro-B-type natriuretic peptide; PAC, premature atrial contraction.

#### Development and validation of the PAVA score

**Figure 1:**
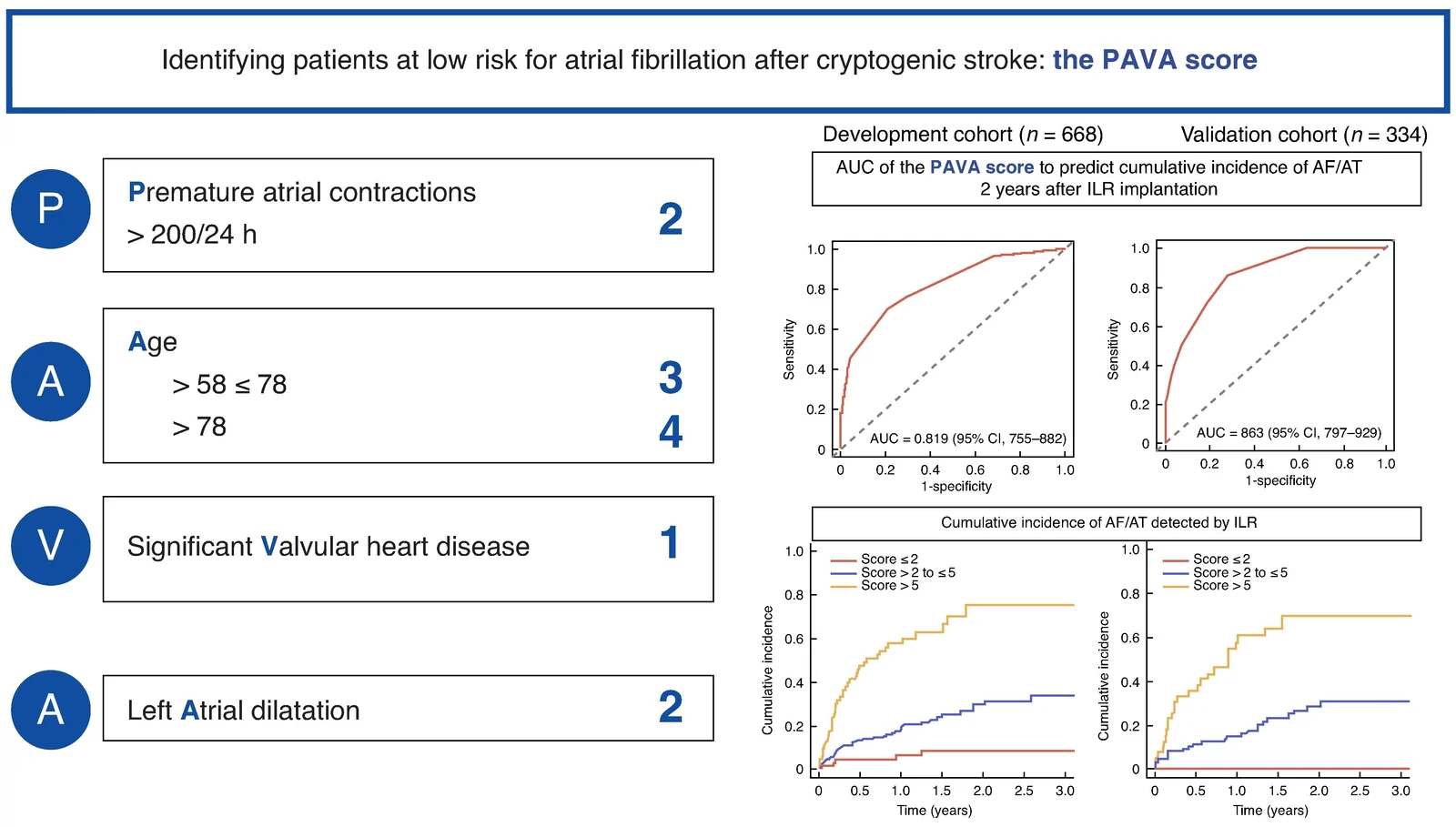
Central illustration of the PAVA score. Components of the PAVA score and their corresponding point values. Area under the curve of the PAVA score in the development and validation cohort. Cumulative incidence of AF in the development and validation cohort according to the PAVA score cutoff. AF, atrial fibrillation; AT, atrial tachycardia; AUC, area under the curve; ILR, implantable loop recorder.

After considering potential confounders, several risk factors remained significantly associated with AF/AT occurrence (*Table 2*). The predictive model developed using the PAVA (premature atrial contractions, age, significant valvular heart disease, and left atrial dilatation) score showed good discrimination in the development cohort (c-index 0.73, 95% CI 0.68–0.77; *n* = 292). The area under the curve (AUC) for the PAVA score in the development cohort was 0.819 (95% CI 0.756–0.882) for AF/AT diagnosis at 2-year follow-up (*Figure 1*).

**Table 2:** Logistic regression model of variables related with cumulative incidence of AF/AT at the end of follow-up, detected by implantable loop recorders after cryptogenic stroke

Variable	Multivariable HR (95% CI) of cumulative incidence of AF/AT over time	*P* value	PAVA score points
Sex (male)	0.71 (0.45–1.14)	0.16	
Hypertension	0.82 (0.51–1.35)	0.44	
Hypercholesterolaemia	1.34 (0.86–2.12)	0.19	
Obese (BMI > 30 kg/m^2^)	1.13 (0.62–2.06)	0.68	
Left atrial dilatation	2.28 (1.50–3.48)	<0.001	2
Significant valvular heart disease	1.83 (1.02–3.26)	0.04	1
Patent foramen ovale	0.84 (0.43–1.65)	0.62	
Left ventricular hypertrophy	0.81 (0.49–1.36)	0.44	
PAC > 200/24 h	2.20 (1.41–3.45)	<0.001	2
Age (years)			
>58 and ≤78	3.94 (1.69–9.21)	0.001	3
>78	6.88 (2.66–17.82)	<0.001	4

AF, atrial fibrillation; AT: atrial tachycardia; BMI, body mass index; CI, confidence interval; HR, hazard ratio; PAC, premature atrial contraction; PAVA, premature atrial contractions, age, significant valvular heart disease, and left atrial dilatation. Number of PAC/24 h were calculated during the initial diagnosis workflow Holter monitoring.

In the validation cohort, 102 (30.5%) patients experienced an AF/AT episode during a mean delay of 252 (262) days after ILR implantation. Evaluation of the predictive value of the PAVA score in the validation cohort showed consistent results, with good predictive accuracy 2 years after ILR implantation (c-index 0.79, 95% CI 0.73–0.85, *n* = 166; AUC 0.863, 95% CI 0.797–0.929) (*Figure 1*).

According to the best accuracy diagnosis value, we defined ≤ 2 as the lower value (low-risk patients) and ≥ 6 as the upper value (high-risk patients) of the PAVA score (see Supplementary material online, *Table S2*). The cumulative incidence of AF/AT detected by ILR after cryptogenic stroke according to the patient’s estimated risk in the development and validation cohorts is illustrated on *Figure 1.*

**Figure 2:**
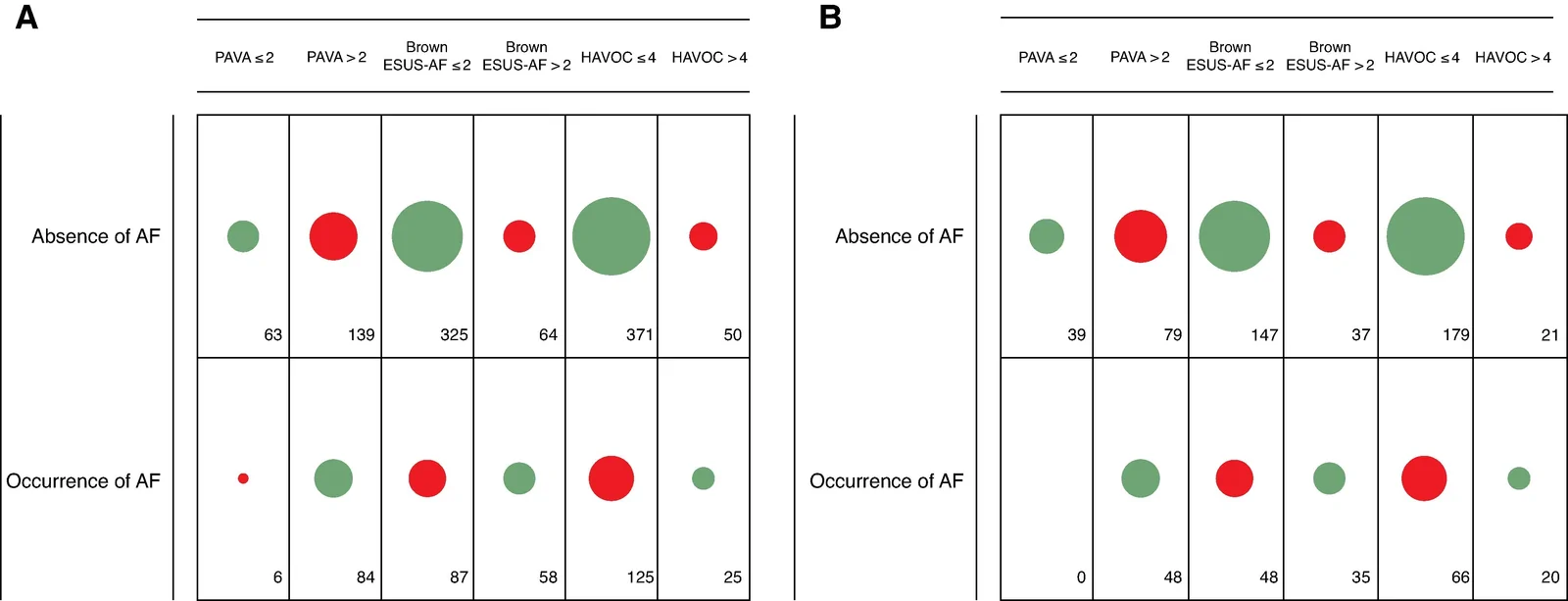
Utility of the PAVA, Brown ESUS-AF, and HAVOC scores in the (*A*) development and (*B*) validation cohorts. AF, atrial fibrillation; ESUS-AF, embolic stroke of undetermined source-atrial fibrillation.

In the development cohort, when calculable, 69/292 (23.6%) patients were considered at low risk of AF/AT. Among these low-risk patients, 6 (8.7%) developed AF/AT by the end of follow-up (*Figure 2A*). The results were similar in the validation cohort, with 39/166 patients (23.5%) at low risk; none developed AF/AT (*Figure 2B*).

### Sensitivity analyses

At 1- and 3-year follow-up after ILR implantation, the AUC for the PAVA score were, respectively, 0.759 (95% CI 0.692–0.827), 0.800 (95% CI 0.717–0.884) in the development cohort. In the validation cohort, AUC were 0.849 (95% CI 0.781–0.917), 0.905 (95% CI 0.836–0.974) at 1- and 3-year follow-up after ILR implantation.

An unweighted model was calculated in the development cohort (*Table 2*). The c-index value was 0.74 (95% CI 0.70–0.79) and the 2-year AUC was 0.825 (95% CI 0.762–0.889) to predict cumulative incidence of AF/AT in the development cohort. The predictive value of the unweighted model was not significantly different from that of the PAVA score (*P* = 0.6).

After multiple imputation of missing data, the performance of our model to predict AF/AT after ILR implantation for cryptogenic stroke remained high in both the development and validation cohorts (c-index 0.70 [95% CI 0.66–0.74] and 0.69 [95% CI 0.64–0.74], respectively). In the development cohort, AUC at 2 years after ILR implantation was 0.74 (95% CI 0.689–0.794). These results were consistent with the validation cohort (0.742, 95% CI 0.670–0.813).

One-hundred and twenty patients were included in the external validation dataset (mean age 67.2 [11.0] years); (see Supplementary material online, *Table S3*). Cumulative incidences of AF/AT at 1, 2, and 3 years were 24.0%, 36.5%, and 39.1%, respectively. In the external validation cohort, the PAVA model had a c-index of 0.66 (95% CI 0.55–0.73) for AF/AT after ILR implantation for cryptogenic stroke. The AUC at 2 years after ILR implantation was 0.697 (95% CI 0.642–0.753).

#### Comparison of PAVA score with the HAVOC and Brown ESUS AF scores

**Figure 3:**
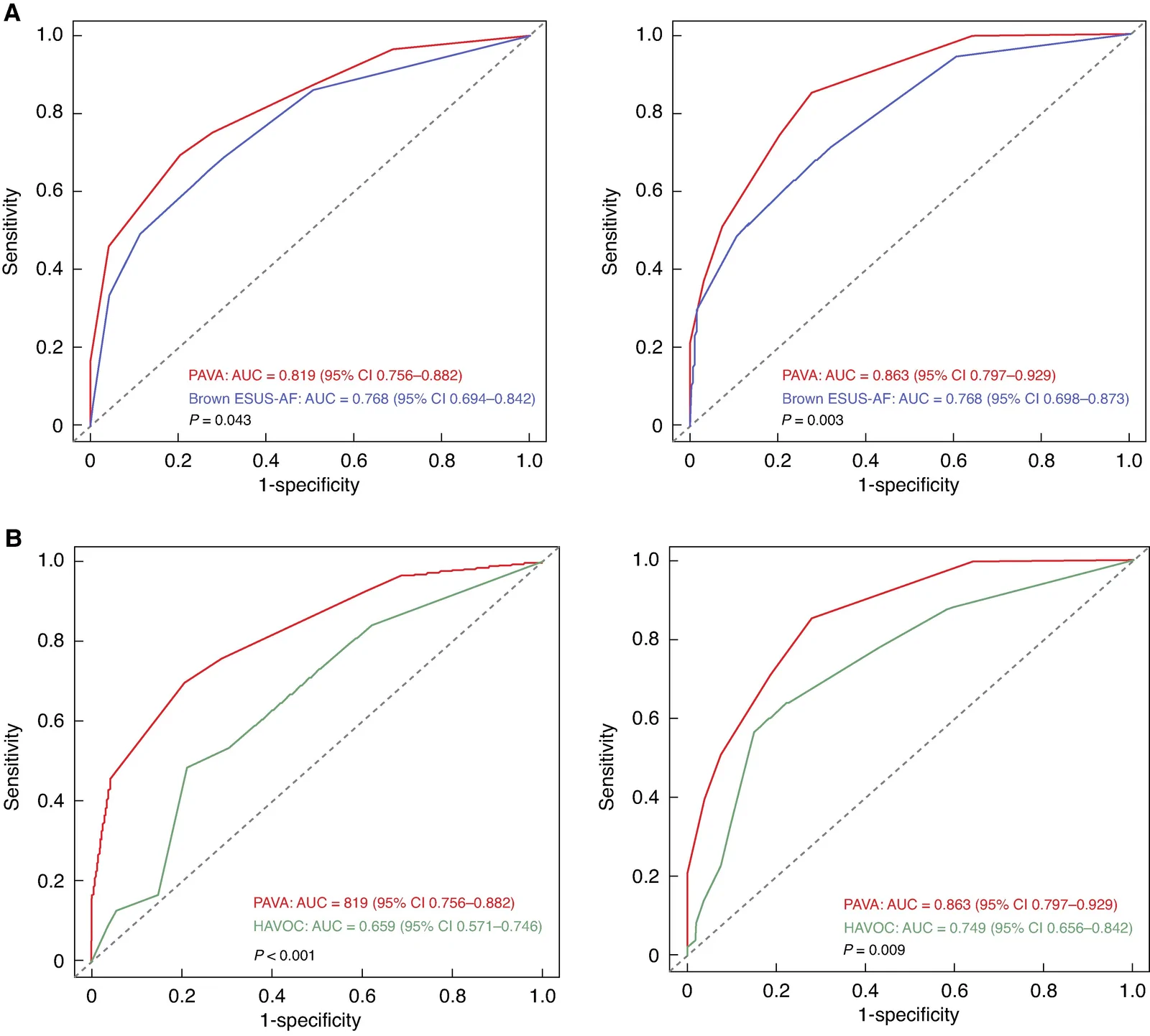
Receiver-operating characteristic curves for the PAVA score compared with (A) the Brown ESUS-AF score and (B) the HAVOC score, 2 years after implantable loop recorder implantation following cryptogenic stroke. In each panel, the development cohort is shown on the left and the validation cohort on the right. Orange curves: PAVA score; blue curves: Brown ESUS-AF score; green curves: HAVOC score. AUC, area under the curve; PAVA, premature atrial contractions, age, valvular heart disease, left atrial dilatation; ROC, receiver-operating characteristic. .

ROC-curve analysis showed that the PAVA score had the best clinical usefulness of the three risk scores tested to predict cumulative incidence of AF/AT 2 years after ILR implantation for cryptogenic stroke (*Figure 3*). As shown in *Figure 2*, 87 (21.1%) patients in the development cohort and 48 (24.6%) in the validation cohort who were initially considered at low risk according to the Brown-ESUS AF score experienced at least one episode of AF/AT during follow-up. Similarly, 125 (25.2%) and 66 (26.9%), respectively, patients considered at low risk of AF/AT according to the HAVOC score experienced at least one episode of AF/AT after a cryptogenic stroke.

## Discussion

Based on a large multicentre cohort of patients with cryptogenic stroke undergoing ILR monitoring, we developed a clinical score designed to estimate the risk of atrial fibrillation after stroke, with particular relevance for identifying patients at lower AF risk, who represented more than one in five of the study population.

Most of the cardiological factors associated with AF (age, female sex, LA dilatation, electrical parameters [*P*-wave duration and force], and heart failure) are related to the presence of atrial cardiomyopathy, which is defined as any structural or electrical alteration of the atria.^31^ Age is a major risk factor for atrial cardiomyopathy and incident AF,^22,32,33^ and has been directly associated with the presence of LA fibrosis, the cornerstone of the development and maintenance of AF.^34^ Both the Brown ESUS-AF and HAVOC scores include an age cutoff. However, based on CHA_2_DS_2_-VASc, these scores used ‘standardized age cutoffs’ of 65 and/or 75 years. In the development of the PAVA score, we chose to assess the best cutoff values for age to predict or rule out AF after cryptogenic stroke, which may have helped to increase the predictive accuracy of the PAVA score relative to existing scores. The highly sensitive cutoff of 10% under the age of 58 years still represented 25.2% of the population, while the highly specific cutoff of 90% over the age of 78 years represented 12.7% of the population.

Left atrial dilatation has been established as a pre-AF stage in the recent Heart Rhythm Society guidelines.^35^ Reflecting structural LA remodelling and increased LA critical mass, LA dilatation has an important electrophysiological impact on AF initiation and maintenance^36^ and constitutes a key feature of atrial cardiomyopathy. Increased LA fibrosis and LA volume have also been associated with a higher risk of thromboembolism in patients with AF.^37–39^ Various measurement strategies and definitions of LA dilatation are currently used in clinical practice, including LA diameter, LA surface area, and LA volume, either indexed to body surface area or not.^40–43^ As in the Brown ESUS-AF score, we incorporated all definitions of LA dilatation into the PAVA score rather than restricting the analysis to more contemporary measurements such as LA volume index. A stricter definition of LA dilatation may have further highlighted the impact of LA dimensions on AF occurrence within the PAVA score. *P*-wave duration could also reflect the underlying atrial cardiomyopathy and might therefore have been a valuable addition to our analysis; however, its reliable measurement requires a dedicated ECG paper speed of 50 mm/s and increased gain (typically doubled to 20 mm/mV), which was not routinely used across our cohort.^44,45^ Overall, the PAVA score reflects the real-world use of LA measurements and the assessment of atrial cardiomyopathy in this large multicentre cohort.

By reflecting various underlying cardiomyopathies, any significant VHD also promotes structural remodelling that could lead to LA dilatation and AF. The presence of VHD has been described as a risk factor for AF after stroke and is also included in the HAVOC score.^13,46^ Interestingly, the predictive value of VHD for AF was not collinear with that of LA dilatation, suggesting that the effect of VHD on AF risk is not solely related to the effect of valvular disease on LA dimensions.

Excessive supraventricular ectopic activity reflects electrical remodelling, which promotes triggers of AF^36,47^ and can also be considered a pre-stage of AF.^35^ Various definitions of excessive supraventricular ectopic activity have been proposed, including different burden cut-offs for PAC, presence, and duration of atrial burst.^48^ Gladstone *et al*. showed that AF occurrence linearly increased with PAC burden documented on ambulatory Holter monitoring.^49^ Similarly, the cut-off of 200 PAC/24 h used in the PAVA score has been previously published.^22^ Twenty-four-hour ECG monitoring is required after all ischaemic strokes to detect in-hospital atrial fibrillation.^5,47^ While Holter monitoring software typically calculates daily PAC counts automatically, most ICU telemetry systems lack the capability to assess PAC burden, which explains the missing data in our cohort. The development of technology to automate the calculation of PAC burden in ICU telemetry could standardize and expand its use.

By incorporating a more granular assessment of PAC burden, the PAVA score enables a more refined stratification of AF risk compared with the HAVOC and Brown-ESUS-AF scores. Patients classified as low risk (PAVA ≤ 2) represented 23.6% of the study population, supporting the potential role of PAVA in risk-based triage and prioritization of ILR implantation. In this group, a cumulative AF/AT incidence of 8.6% was observed in the development cohort, with no AF/AT events detected in the validation cohort by the end of follow-up. These rates are broadly comparable to those reported in the general population^50,51^ and after non-cardioembolic stroke^52^ undergoing ILR monitoring. Importantly, patients classified as low risk represented 23.6% of the study population, suggesting that the PAVA score may support risk-based ILR implantation in more than one in five post-stroke patients. Based on these findings, further prospective studies are needed to assess the clinical impact of PAVA-guided prioritization of ILR implantation on outcomes such as recurrent stroke, anticoagulation decisions, bleeding risk, quality of life, and cost-effectiveness, particularly given the uncertain imputability of AF detected after the initial stroke.^53^

Other scores^16,54,55^ with good predictive accuracy for AF after stroke have been published. The STAF score demonstrated very high predictive value of AF after stroke (AUC 0.94).^54^ However, it was based on AF diagnosed during hospital stay, before the diagnosis of cryptogenic stroke, and thus does not apply to our population of ILR-implanted patients with cryptogenic stroke. The SURF score included AF diagnosed during hospitalization (thus initially classified as cardioembolic strokes) as well as during 2-year follow-up (classified as cryptogenic strokes), using external Holter monitoring and repeated ECG after stroke. This explains the lower prevalence of AF in their study (14%) compared with studies using ILR. Therefore, the SURF score cannot be applied to patients with cryptogenic stroke alone due to population heterogeneity. The C_2_HEST score has also shown promising results in predicting AF after stroke,^56^ a finding that was subsequently confirmed in a cohort of cryptogenic stroke patients undergoing ILR monitoring.^18^ Unfortunately, chronic obstructive pulmonary disease and thyroid disease were not systematically collected in our cohort, resulting in a substantial amount of missing data that precluded meaningful analysis of these variables. Further studies are warranted to evaluate the incremental diagnostic yield of incorporating these variables into the PAVA score. Finally, PROACTIA^15^ showed a similar predictive value (AUC 0.79) to PAVA for predicting AF after cryptogenic stroke using ILR. Nevertheless, until now, no external validation has been performed. Furthermore, the PROACTIA score was calculated from an unweighted formula, making it impossible to calculate at the patient’s bedside, and the speed of 50 mm/s for the baseline ECG limits its use in daily practice. In contrast, we aimed to develop a score that is easy to memorize and calculate, without complex formulae or rare variables, with the goal of assisting clinicians in the decision regarding ILR implantation after cryptogenic stroke.

### Limitations

A main limitation is inherent to the observational nature of this study, which includes potential biases. This limitation is partly countered by the large number of patients included. In France, unlike many other European countries, reimbursement for post-stroke ILR in adults is not subject to selection criteria such as age and NT-proBNP value. Thus, we were able to include a large population that was representative of patients with cryptogenic stroke treated in tertiary centres without the bias of prespecified implantation criteria.

Diagnosis of AF/AT is based on ILR alerts. Only available episodes were reviewed, on selected platforms, and depend on embedded algorithms that vary by type of ILR. Previous studies have also highlighted the value of implanting ILRs within the first weeks following cryptogenic stroke or during hospitalization period.^52,57^ This may have led to an underestimation of AF/AT occurrence. However, the incidence rate of AF/AT in our study is similar to previous reports of AF incidence following cryptogenic stroke.^1,2,15^ Moreover, AF detection in our study relied on a dichotomous approach (AF present/absent), partly because the embedded algorithms of the ILRs currently do not provide reliable automated quantification of AF burden or episode duration. Although the concept of AF burden has recently been proposed by the ESC Council on Stroke and the EHRA as a more granular metric for AF quantification,^58^ such data were not available in our multicentre cohort. Future studies on post-stroke AF detection should therefore consider integrating AF burden assessment to refine risk stratification and guide therapeutic decision-making.

Missing data are inherent to large population studies. Complete-case analysis was used for model development, rather than imputation, and may have affected estimates of the overall effect size. To account for this limitation, we calculated the predictive value of our score after multiple imputation. The results showed similar predictive values in both the development and validation cohorts.

## Conclusions

The PAVA score, developed and validated in a large multicentre French cohort, provides a targeted assessment of AF risk after cryptogenic stroke in patients undergoing ILR monitoring. It may support risk-adapted diagnostic and therapeutic strategies and enable more efficient allocation of cardiac monitoring resources, although prospective studies are required to establish the clinical and health-economic impact of PAVA-guided approaches.

## Supplementary Material

euag183_Supplementary_Data

## Data Availability

Data supporting the findings of this study are available from the corresponding author upon reasonable request.

## References

[1] Brachmann J, Morillo CA, Sanna T, Di Lazzaro V, Diener HC, Bernstein RA et al Uncovering atrial fibrillation beyond short-term monitoring in cryptogenic stroke patients: three-year results from the cryptogenic stroke and underlying atrial fibrillation trial. Circ Arrhythm Electrophysiol 2016;9:e003333. 26763225 10.1161/CIRCEP.115.003333

[2] Buck BH, Hill MD, Quinn FR, Butcher KS, Menon BK, Gulamhusein S et al Effect of implantable vs prolonged external electrocardiographic monitoring on atrial fibrillation detection in patients with ischemic stroke: the PER DIEM randomized clinical trial. JAMA 2021;325:2160–8. 34061146 10.1001/jama.2021.6128 PMC8170545

[3] Sanna T, Diener HC, Passman RS, Lazzaro D, Bernstein V, Morillo RA et al Cryptogenic stroke and underlying atrial fibrillation. N Engl J Med 2014;370:2478–86. 24963567 10.1056/NEJMoa1313600

[4] Van Gelder IC, Rienstra M, Bunting KV, Casado-Arroyo R, Caso V, Crijns HJGM et al 2024 ESC guidelines for the management of atrial fibrillation developed in collaboration with the European Association for Cardio-Thoracic Surgery (EACTS). Eur Heart J 2024;45:3314–414. 39210723 10.1093/eurheartj/ehae176

[5] Rubiera M, Aires A, Antonenko K, Lémeret S, Nolte CH, Putaala J et al European Stroke Organisation (ESO) guideline on screening for subclinical atrial fibrillation after stroke or transient ischaemic attack of undetermined origin. Eur Stroke J 2022;7:CVII–CXXXIX. 10.1177/23969873221099478 PMC9446336 36082257

[6] Rienstra M, Tzeis S, Bunting KV, Caso V, Crijns HJGM, De Potter TJR et al Spotlight on the 2024 ESC/EACTS management of atrial fibrillation guidelines: 10 novel key aspects. Europace 2024;26:euae298. 39716733 10.1093/europace/euae298 PMC11666470

[7] Meynet I, Karvonen J, Boriani G, Penela D, Mazurek M, Mugnai G et al Management of device-detected subclinical atrial fibrillation: a European Heart Rhythm Association survey. Europace 2025;27:euaf284. 41206582 10.1093/europace/euaf284 PMC12677020

[8] Samuel M, Van Der Stoel M, Van Dijk V, Maass A. Real-world evaluation of the treatment yield of implantable loop recorders (ILR) in the Netherlands. Europace 2024;26:euae102-666.

[9] Béjot Y, Duloquin G, Guenancia C. Incidence and nationwide estimates of cryptogenic ischemic stroke or TIA eligible for prolonged cardiac rhythm monitoring. Eur Stroke J 2024;10:153–60. 39109522 10.1177/23969873241266471 PMC11569448

[10] Dilaveris PE, Antoniou CK, Caiani EG, Casado-Arroyo R, Climent AΜ, Cluitmans M et al ESC Working Group on e-Cardiology Position Paper: accuracy and reliability of electrocardiogram monitoring in the detection of atrial fibrillation in cryptogenic stroke patients. Eur Heart J Digit Health 2022;3:341–58. 36712155 10.1093/ehjdh/ztac026 PMC9707962

[11] Alonso A, Krijthe BP, Aspelund T, Stepas KA, Pencina MJ, Moser CB et al Simple risk model predicts incidence of atrial fibrillation in a racially and geographically diverse population: the CHARGE-AF consortium. J Am Heart Assoc 2013;2:e000102. 23537808 10.1161/JAHA.112.000102 PMC3647274

[12] Li YG, Pastori D, Farcomeni A, Yang PS, Jang E, Joung B et al A simple clinical risk score (C2HEST) for predicting incident atrial fibrillation in Asian subjects: derivation in 471,446 Chinese subjects, with internal validation and external application in 451,199 Korean subjects. Chest 2019;155:510–8. 30292759 10.1016/j.chest.2018.09.011 PMC6437029

[13] Kwong C, Ling AY, Crawford MH, Zhao SX, Shah NH. A clinical score for predicting atrial fibrillation in patients with cryptogenic stroke or transient ischemic attack. Cardiology 2017;138:133–40. 28654919 10.1159/000476030 PMC5683906

[14] Ricci B, Chang AD, Hemendinger M, Dakay K, Cutting S, Burton T et al A simple score that predicts paroxysmal atrial fibrillation on outpatient cardiac monitoring after embolic stroke of unknown source. J Stroke Cerebrovasc Dis 2018;27:1692–6. 29501269 10.1016/j.jstrokecerebrovasdis.2018.01.028

[15] Skrebelyte-Strøm L, Rønning OM, Dahl FA, Steine K, Kjekshus H. Prediction of occult atrial fibrillation in patients after cryptogenic stroke and transient ischaemic attack: PROACTIA. Europace 2022;24:1881–8. 35819199 10.1093/europace/euac092 PMC9733955

[16] Ratajczak-Tretel B, Lambert AT, Al-Ani R, Arntzen K, Bakkejord GK, Bekkeseth HMO et al Prediction of underlying atrial fibrillation in patients with a cryptogenic stroke: results from the NOR-FIB study. J Neurol 2023;270:4049–59. 37162578 10.1007/s00415-023-11680-8 PMC10344988

[17] Grygorowicz C, Benali K, Serzian G, Mouhat B, Duloquin G, Pommier T et al Value of HAVOC and Brown ESUS-AF scores for atrial fibrillation on implantable cardiac monitors after embolic stroke of undetermined source. J Stroke Cerebrovasc Dis 2024;33:107451. 37995501 10.1016/j.jstrokecerebrovasdis.2023.107451

[18] Palaiodimou L, Theodorou A, Triantafyllou S, Dilaveris P, Flevari P, Giannopoulos G et al Performance of different risk scores for the detection of atrial fibrillation among patients with cryptogenic stroke. Stroke 2024;55:454–62. 38174570 10.1161/STROKEAHA.123.044961

[19] Zhu W, Wan R, Liu F, Hu J, Huang L, Li J et al Relation of body mass index with adverse outcomes among patients with atrial fibrillation: a meta-analysis and systematic review. J Am Heart Assoc 2016;5:e004006. 27613773 10.1161/JAHA.116.004006 PMC5079045

[20] Vahanian A, Beyersdorf F, Praz F, Milojevic M, Baldus S, Bauersachs J et al 2021 ESC/EACTS guidelines for the management of valvular heart disease: developed by the task force for the management of valvular heart disease of the European Society of Cardiology (ESC) and the European Association for Cardio-Thoracic Surgery (EACTS). Eur Heart J 2022;43:561–632. 35636831 10.1016/j.rec.2022.05.006

[21] Goette A, Kalman JM, Aguinaga L, Akar J, Cabrera JA, Chen SA et al EHRA/HRS/APHRS/SOLAECE expert consensus on atrial cardiomyopathies: definition, characterization, and clinical implication. Europace 2016;18:1455–90. 27402624 10.1093/europace/euw161 PMC6392440

[22] Shimada Y, Todo K, Doijiri R, Yamazaki H, Sonoda K, Koge J et al Higher frequency of premature atrial contractions correlates with atrial fibrillation detection after cryptogenic stroke. Stroke 2024;55:946–53. 38436115 10.1161/STROKEAHA.123.044813

[23] Prentice RL, Kalbfleisch JD, Peterson AV Jr, Flournoy N, Farewell VT, Breslow NE. The analysis of failure times in the presence of competing risks. Biometrics 1978;34:541–54. 373811

[24] Wolbers M, Koller MT, Witteman JCM, Steyerberg EW. Prognostic models with competing risks: methods and application to coronary risk prediction. Epidemiology 2009;20:555–61. 19367167 10.1097/EDE.0b013e3181a39056

[25] Fine JP, Gray RJ. A proportional hazards model for the subdistribution of a competing risk. J Am Stat Assoc 1999;94:496.

[26] Harrell FE, Lee KL, Mark DB. Multivariable prognostic models: issues in developing models, evaluating assumptions and adequacy, and measuring and reducing errors. Stat Med 1996;15:361–87. 8668867 10.1002/(SICI)1097-0258(19960229)15:4<361::AID-SIM168>3.0.CO;2-4

[27] Gray RJ . A class of K-sample tests for comparing the cumulative incidence of a competing risk. Ann Stat 1988;16:1141–54.

[28] Little RJA, Rubin DB. Statistical Analysis with Missing Data. 2nd ed. Hoboken, NJ: Wiley; 2002.

[29] White IR, Royston P, Wood AM. Multiple imputation using chained equations: issues and guidance for practice. Stat Med 2011;30:377–99. 21225900 10.1002/sim.4067

[30] Rubin DB . Multiple Imputation for Nonresponse in Surveys. 1st ed. New York, NY: Wiley; 1987.

[31] Goette A, Corradi D, Dobrev D, Aguinaga L, Cabrera JA, Chugh SS et al Atrial cardiomyopathy revisited—evolution of a concept. A clinical consensus statement of the European Heart Rhythm Association (EHRA) of the ESC, the Heart Rhythm Society (HRS), the Asian Pacific Heart Rhythm Association (APHRS), and the Latin American Heart Rhythm Society (LAHRS). Europace 2024;26:euae204. 39077825 10.1093/europace/euae204 PMC11431804

[32] Kistler PM, Sanders P, Fynn SP, Stevenson IH, Spence SJ, Vohra JK et al Electrophysiologic and electroanatomic changes in the human atrium associated with age. J Am Coll Cardiol 2004;44:109–16. 15234418 10.1016/j.jacc.2004.03.044

[33] Roberts-Thomson KC, Kistler PM, Sanders P, Morton JB, Haqqani HM, Stevenson I et al Fractionated atrial electrograms during sinus rhythm: relationship to age, voltage, and conduction velocity. Heart Rhythm 2009;6:587–91. 19329365 10.1016/j.hrthm.2009.02.023

[34] Shen MJ, Arora R, Jalife J. Atrial myopathy. JACC Basic Transl Sci 2019;4:640–54. 31768479 10.1016/j.jacbts.2019.05.005 PMC6872845

[35] Joglar JA, Chung MK, Armbruster AL, Benjamin EJ, Chyou JY, Cronin EM et al 2023 ACC/AHA/ACCP/HRS guideline for the diagnosis and management of atrial fibrillation: a report of the American College of Cardiology/American Heart Association joint committee on clinical practice guidelines. Circulation 2024;149:e1–156. 38033089 10.1161/CIR.0000000000001193 PMC11095842

[36] Maille B, Lalevée N, Marlinge M, Vahdat J, Mottola G, Degioanni C et al Adenosine and adenosine receptors: advances in atrial fibrillation. Biomedicines 2022;10:2963. 36428533 10.3390/biomedicines10112963 PMC9687155

[37] Hirsh Benjamin J, Copeland-Halperin Robert S, Halperin Jonathan L. Fibrotic atrial cardiomyopathy, atrial fibrillation, and thromboembolism. J Am Coll Cardiol 2015;65:2239–51. 25998669 10.1016/j.jacc.2015.03.557

[38] Daccarett M, Badger TJ, Akoum N, Burgon NS, Mahnkopf C, Vergara G et al Association of left atrial fibrosis detected by delayed-enhancement magnetic resonance imaging and the risk of stroke in patients with atrial fibrillation. J Am Coll Cardiol 2011;57:831–8. 21310320 10.1016/j.jacc.2010.09.049 PMC3124509

[39] Goette A, Lemoine MD, Borof K, Schotten U, Breithardt G, Camm AJ et al Prevalence and severity of atrial cardiomyopathy in patients with recently diagnosed atrial fibrillation and stroke risk factors and its association with early rhythm control: a secondary analysis of EAST-AFNET 4. Europace 2025;27:euaf256. 41061672 10.1093/europace/euaf256 PMC12559886

[40] Ntaios G, Baumgartner H, Doehner W, Donal E, Edvardsen T, Healey JS et al Embolic strokes of undetermined source: a clinical consensus statement of the ESC Council on Stroke, the European Association of Cardiovascular Imaging and the European Heart Rhythm Association of the ESC. Eur Heart J 2024;45:1701–15. 38685132 10.1093/eurheartj/ehae150 PMC11107123

[41] Healey JS, Gladstone DJ, Swaminathan B, Eckstein J, Mundl H, Epstein AE et al Recurrent stroke with rivaroxaban compared with aspirin according to predictors of atrial fibrillation: secondary analysis of the NAVIGATE ESUS randomized clinical trial. JAMA Neurol 2019;76:764–73. 30958508 10.1001/jamaneurol.2019.0617 PMC6583060

[42] Kamel H, Longstreth WT, Tirschwell DL, Kronmal RA, Marshall RS, Broderick JP et al Apixaban to prevent recurrence after cryptogenic stroke in patients with atrial cardiopathy: the ARCADIA randomized clinical trial. JAMA 2024;331:573–81. 38324415 10.1001/jama.2023.27188 PMC10851142

[43] McIntyre WF, Benz AP, Becher N, Healey JS, Granger CB, Rivard L et al Direct oral anticoagulants for stroke prevention in patients with device-detected atrial fibrillation: a study-level meta-analysis of the NOAH-AFNET 6 and ARTESiA trials. Circulation 2024;149:981–8. 37952187 10.1161/CIRCULATIONAHA.123.067512

[44] Chen LY, Ribeiro ALP, Platonov PG, Cygankiewicz I, Soliman EZ, Gorenek B et al P wave parameters and indices: a critical appraisal of clinical utility, challenges, and future research—a consensus document endorsed by the International Society of Electrocardiology and the International Society for Holter and Noninvasive Electrocardiology. Circ Arrhythm Electrophysiol 2022;15:e010435. 35333097 10.1161/CIRCEP.121.010435 PMC9070127

[45] Didier R, Garnier L, Duloquin G, Meloux A, Sagnard A, Graber M et al Distribution of atrial cardiomyopathy markers and association with atrial fibrillation detected after ischaemic stroke in the SAFAS study. Stroke Vasc Neurol 2024;9:165–73. 37429637 10.1136/svn-2023-002447 PMC11103154

[46] Poh MQW, Tham CH, Chee JDMS, Saffari SE, Tan KWK, Tan LW et al Predicting atrial fibrillation after ischemic stroke: clinical, genetics, and electrocardiogram modelling. Cerebrovasc Dis Extra 2022;13:9–17. 36521445 10.1159/000528516 PMC10015706

[47] Snyman S, Seder E, David-Muller M, Klein V, Doche E, Suissa L et al Atrial fibrillation detected by implantable monitor in embolic stroke of undetermined source: a new clinical entity. J Clin Med 2022;11:5740. 36233608 10.3390/jcm11195740 PMC9571950

[48] Larsen BS, Kumarathurai P, Falkenberg J, Nielsen OW, Sajadieh A. Excessive atrial ectopy and short atrial runs increase the risk of stroke beyond incident atrial fibrillation. J Am Coll Cardiol 2015;66:232–41. 26184616 10.1016/j.jacc.2015.05.018

[49] Gladstone DJ, Dorian P, Spring M, Panzov V, Mamdani M, Healey JS et al Atrial premature beats predict atrial fibrillation in cryptogenic stroke. Stroke 2015;46:936–41. 25700289 10.1161/STROKEAHA.115.008714

[50] Svendsen JH, Diederichsen SZ, Højberg S, Krieger DW, Graff C, Kronborg C et al Implantable loop recorder detection of atrial fibrillation to prevent stroke (the LOOP study): a randomised controlled trial. Lancet 2021;398:1507–16. 34469766 10.1016/S0140-6736(21)01698-6

[51] Svennberg E, Friberg L, Frykman V, Al-Khalili F, Engdahl J, Rosenqvist M. Clinical outcomes in systematic screening for atrial fibrillation (STROKESTOP): a multicentre, parallel group, unmasked, randomised controlled trial. Lancet 2021;398:1498–506. 34469764 10.1016/S0140-6736(21)01637-8

[52] Bernstein RA, Kamel H, Granger CB, Piccini JP, Sethi PP, Katz JM et al Effect of long-term continuous cardiac monitoring vs usual care on detection of atrial fibrillation in patients with stroke attributed to large- or small-vessel disease: the STROKE-AF randomized clinical trial. JAMA 2021;325:2169–77. 34061145 10.1001/jama.2021.6470 PMC8170544

[53] Sposato LA, Field TS, Schnabel RB, Wachter R, Andrade JG, Hill MD. Towards a new classification of atrial fibrillation detected after a stroke or a transient ischaemic attack. Lancet Neurol 2024;23:110–22. 37839436 10.1016/S1474-4422(23)00326-5

[54] Suissa L, Bertora D, Lachaud S, Mahagne MH. Score for the targeting of atrial fibrillation (STAF): a new approach to the detection of atrial fibrillation in the secondary prevention of ischemic stroke. Stroke 2009;40:2866–8. 19461041 10.1161/STROKEAHA.109.552679

[55] Suissa L, Bertora D, Kalle R, Bruno C, Romero G, Mahagne MH. SURF (stroke with underlying risk of atrial fibrillation): proposals for a definition. Clin Neurol Neurosurg 2019;182:43–8. 31078954 10.1016/j.clineuro.2019.04.028

[56] Li Y, Bisson A, Bodin A, Herbert J, Grammatico-Guillon L, Joung B et al C2 HEST score and prediction of incident atrial fibrillation in poststroke patients: a French nationwide study. J Am Heart Assoc 2019;8:e012546. 31234697 10.1161/JAHA.119.012546 PMC6662366

[57] Milstein NS, Musat DL, Allred J, Seiler A, Pimienta J, Oliveros S et al Detection of atrial fibrillation using an implantable loop recorder following cryptogenic stroke: implications for post-stroke electrocardiographic monitoring. J Interv Card Electrophysiol 2020;57:141–7. 31612300 10.1007/s10840-019-00628-6

[58] Doehner W, Boriani G, Potpara T, Blomstrom-Lundqvist C, Passman R, Sposato LA et al Atrial fibrillation burden in clinical practice, research, and technology development: a clinical consensus statement of the European Society of Cardiology Council on Stroke and the European Heart Rhythm Association. Europace 2025;27:euaf019. 40073206 10.1093/europace/euaf019 PMC11901050

