# Supplementary material for: Identifying patients at low risk for atrial fibrillation after cryptogenic stroke: the PAVA score
Source: Europace. 2026 Sep 3;28(9):euag183. doi: 10.1093/europace/euag183 (PMC13549145; doi:10.1093/europace/euag183)
Supplement: Supplementary file 1 [file euag183_supplementary_data.docx]

**Supplementary Table 1 : Baseline characteristics in the development and validation cohort**

|  | Development cohort (n=668) | Validation cohort (n=334) | p value |
| --- | --- | --- | --- |
| Hospital of origin |  |  | 0.5192 |
| - BESANCON | 41(6.1) | 12(3.6) |  |
| - DIJON | 216(32.3) | 113(33.8) |  |
| - MARSEILLE | 174(26.1) | 89(26.7) |  |
| - PARIS | 161(24.1) | 85(25.4) |  |
| - RENNES | 76(11.4) | 35(10.5) |  |
| Age (mean± SD) | 65.7±12.2 | 67.2±12.4 | 0.069 |
| Male Sex | 387(57.9) | 193(77.8) | 0.9639 |
| Stroke localization |  |  | 0.0301 |
| - Supra tentorial | 462(71.5) | 201(63.2) |  |
| - Infra tentorial | 151(23.4) | 98(30.8) |  |
| - Both | 33(5.1) | 19(6.0) |  |
| Obesity | 109(17.8) | 55(17.7) | 0.617 |
| Hypertension | 392(58.7) | 189(56.6) | 0.5263 |
| Hypercholesterolemia | 263(39.4) | 134(40.1) | 0.8335 |
| Diabetes mellitus | 100(15.0) | 51(15.3) | 0.9006 |
| Tobacco use | 216(32.3) | 108(32.3) | 1.0000 |
| Alcool intake | 44(9.9) | 28(12.7) | 0.2675 |
| Obstructive sleep apnea | 45(6.8) | 22(6.6) | 0.9193 |
| Peripheral arteriopathy or coronary artery disease | 119(17.8) | 44(13.2) | 0.0606 |
| Active neoplasia | 13(2.2) | 8(2.7) | 0.6614 |
| Heart failure | 12(1.8) | 7(2.1) | 0.7432 |
| CHA^2^DS^2^-VASc score |  |  | 0.5993 |
| - 2 | 75(11.2) | 43(12.9) |  |
| - 3 | 160(23.9) | 73(21.9) |  |
| - 4 | 160(23.9) | 68(20.4) |  |
| - 5 | 142((21.3) | 79(36.6) |  |
| - 6 | 105(15.7) | 52(15.6) |  |
| - 7 | 22(3.3) | 16(4.8) |  |
| - 8 | 3(0.5) | 3(0.9) |  |
| - 9 | 1(0.1) | 0(0) |  |
| LA measurements |  |  |  |
| - LA indexed volume (ml/m²) - LA dilatation | 31.1±12.3  189(35.4) | 31.9±13.3  100(37.5) | 0.45  0.5671 |
| Valvular heart disease | 49(7.9) | 27(8.8) | 0.6465 |
| Left Ventricular hypertrophy | 100(18.8) | 61(22.6) | 0.2002 |
| PFO | 115(19.7) | 35(12.2) | 0.0055 |
| PAC > 200 per 24 hours | 100(26.6) | 64(31.7) | 0.1958 |
| Remote monitoring follow-up | 428(72.3) | 204(68.2) | 0.2065 |
| Occurrence of AF |  |  | 0.1259 |
| - No | 482(72.2) | 230(68.9) |  |
| - Yes | 174(26.0) | 102(30.5) |  |
| - deaths | 12(0.2) | 2(0.6) |  |
| Follow-up. days (mean±SD) | 604±445 | 605±442 | 0.97 |
| Implantable loop Recorder   - Medtronic (Reveal XT 9529 or Reveal LINQ LNQ11) - Abbott (Confirm Rx DM3500) - Biotronik (Biomonitor IIIm 450218) | 549(82.4)  113(17.0)  4(0.6) | 271(81.4)  58(17.4)  4(1.2) | 0.61 |

**Supplemetary table 2: Predictive accuracy of cumulative incidence of AF/AT 2 years after implantable loop recorder implantation for cryptogenic stroke using the PAVA score in the low-risk and high-risk groups**

|  | Low-risk patients (PAVA score ≤2) | | High-risk patients (PAVA ≥6) | |
| --- | --- | --- | --- | --- |
|  | **Development cohort** | **Validation cohort** | **Development cohort** | **Validation cohort** |
| Sensitivity | 94.3 (2.5) | 100 (0) | 45.7 (5.6) | 50.8 (7.4) |
| Specificity | 36.6(5.7) | 35.2 (6.5) | 95.7 (2.4) | 92.6 (3.6) |
| PPV | 42.8 (4.2) | 41.2 (5.0) | 84.8 (7.6) | 75.6 (9.8) |
| NPV | 92.6 (3.3) | 100 (0) | 76.0 (3.2) | 80.6 (3.9) |

AF, atrial fibrillation; AT: atrial tachycardia; NPV, negative predictive value; PAVA, age, significant valvular heart disease, and left atrial appendage dilatation; PPV, positive predictive value.

**Supplementary Table 3: Baseline characteristics in the external validation cohort**

|  | Validation cohort (n=120) |
| --- | --- |
| Age (mean± SD) | 67.2±10.1 |
| Male Sex | 62(51.7) |
| Obesity (BMI> 30) | 25(20.8) |
| Hypertension | 68(57.8) |
| Hypercholesterolemia | 45(37.5) |
| Diabetes mellitus | 18(15) |
| Tobacco use | 39(32.5) |
| Alcool intake | 8(6.7) |
| Obstructive sleep apnea | 21(17.5) |
| Peripheral arteriopathy or coronary artery disease | 14(11.7) |
| Active neoplasia | 6(5.0) |
| Heart failure | 1(0.8) |
| CHA^2^DS^2^-VASc score |  |
| - 2 | 16(13.3) |
| - 3 | 21(17.5) |
| - 4 | 35(29.2) |
| - 5 | 24(20.0) |
| - 6 | 17(14.2) |
| - 7 | 6(5) |
| - 8 | 1(0.8)) |
| - 9 | 0(0) |
| LA measurements |  |
| - LA indexed volume (ml/m²) - LA dilatation | 31.1±12.3  45(37.5) |
| Valvular heart disease | 9(7.5) |
| Left Ventricular hypertrophy | 13(10.8) |
| PFO | 33(27.5) |
| PAC > 200 per 24 hours | 38(31.7) |
| Remote monitoring follow-up | 109(90.8) |
| Occurrence of AF |  |
| - No | 75(62.5) |
| - Yes | 42(35.0) |
| - deaths | 3(2.5) |
| Follow-up. days (mean±SD) | 719±482 |

**Supplementary Figure 1: Spline regression of relationship between age (panel A), number of premature atrial complex per 24h (panel B) with atrial fibrillation risk**

A.

B.
